# Social Drivers of Health and Communication Interventions Impact Wound Care Follow-Up Adherence: A Retrospective Cohort Study at a Tertiary Care Center

**DOI:** 10.3390/clinpract16020042

**Published:** 2026-02-18

**Authors:** Adrian C. Chen, Amit S. Rao, Alisha Oropallo

**Affiliations:** 1 Departments of Medicine and Cardiology, Donald and Barbara Zucker School of Medicine at Hofstra/Northwell, Hempstead, NY 11549, USA; achen7@pride.hofstra.edu; 2 Northwell Health Comprehensive Wound Healing Center & Hyperbarics, Lake Success, New Hyde Park, NY 11030, USA; arao3@northwell.edu

**Keywords:** chronic wound care, follow-up adherence, social drivers of health, hospital readmission, patient decision-making, skilled nursing facilities, wound care outcomes

## Abstract

Introduction: Chronic wounds affect approximately six million people in the United States. Despite established multidisciplinary wound care protocols, patient adherence to follow-up care remains suboptimal. We aimed to understand the impact of social drivers of health on patient decision-making for improving wound care follow-up adherence. Methods: We conducted a retrospective review of all hospitalized patients who consulted in-house wound care staff at a tertiary care center between August 2017 and June 2020, regardless of primary admission diagnosis. Referred patients received standardized care from a multidisciplinary team at an outpatient wound care facility. Primary endpoints were pre-discharge scheduling and follow-up rates. Follow-up efficacy was assessed through 90-day hospital readmission rates. Results: Of 444 patients, 205 (46.2%) were readmitted or expired within 90 days. Adjusted analysis identified lack of follow-up care reception as an independent predictor of hospital readmission (hazard ratio 2.39; 95% CI, 1.45–3.89; *p* < 0.001). Among 156 (35.1%) patients who scheduled follow-up, 110 (70.5%) adhered to their appointment. Patients not scheduling follow-up were older (median age 79 vs. 70 years, *p* < 0.001), longer hospital stays (median 9 vs. 6 days, *p* < 0.0001), and more frequently discharged to skilled nursing facilities (47.6% vs. 26.3%, *p* < 0.0001). Among scheduled patients, skilled nursing home residents demonstrated lower follow-up adherence (OR 0.3; 95% CI, 0.14–0.65; *p* < 0.01). Conclusions: Pre-hospital discharge communication for scheduling follow ups serves as a critical intervention point in patient decision-making for wound follow-up. Considering the limitations of a retrospective single-center study, we find that pre-discharge education about follow-up scheduling for high-risk groups, including patients ≥ 80 years and skilled nursing facility residents, may improve follow-up adherence and reduce readmissions.

## 1. Introduction

Chronic wounds afflict an estimated 2% of the United States population [1]. Due to the aging population and increasing prevalence of diabetes mellitus and cardiovascular disease, the number of Americans with chronic wounds continues to grow. Etiologies behind chronic wounds can arise from multiple origins, including diabetes, venous disease, pressure, surgical, and trauma. This pathology significantly decreases the quality of life via inducing pain, sleep disturbances, mental frustration, and inhibiting activities of daily living [2].

Because the nature of the pathology generally arises from deficits in multiple organ systems (e.g., poor tissue perfusion and immunological response), the interdisciplinary approach provided by wound care centers has proved to be very effective in treating chronic wounds. These centers are made up of comprehensive teams which consist of physicians of numerous specialties, nurses, medical assistants, physical therapists, nutritionists, social workers, orthotists, and administrators [3]. Patients experience reduced pain and complications when treated by a specialized team at a wound care center as opposed to those who are not [4]. Research has concluded that wound centers improve healing rates of chronic wounds [5]. It has also been noted that patients under the care of an interdisciplinary team experience fewer inpatient hospitalizations and limb amputations [6].

Numerous studies have demonstrated the efficacy of having standardized, multidisciplinary wound care during an outpatient follow-up [7,8,9]; however, these outcomes cannot be attained without follow-up adherence. Multiple factors affecting post-discharge adherence to visiting a wound care center exist, including social drivers of health and clinical factors. Examples include insurance type, comorbidities, driving distance to the clinic, as well as many other possibilities. Among patients who have endured traumatic injury, those with Medicaid or without insurance were found to be significantly less likely to attend outpatient follow-up visits than those with private insurance [5]. Socioeconomic or clinical factors associated with post-hospitalization follow-up have not yet been identified in chronic wound patients. Identifying social drivers of health and clinical factors influencing patient follow-up adherence to outpatient wound care professionals may enable targeted interventions for susceptible populations.

This study is grounded in the Behavioral Model of Health Services Use, which posits that healthcare utilization is influenced by predisposing factors (demographics, social structure), enabling factors (resources, access), and need factors (perceived and evaluated health status) [10]. Within this framework, we conceptualize follow-up adherence as a health behavior influenced by social drivers of health—the conditions in which people are born, grow, live, work, and age that shape health outcomes. Following Costa et al.’s (2023) systematic framework for analyzing social drivers in vascular disease populations, we distinguish between structural drivers (socioeconomic position, systemic healthcare barriers) and intermediate factors (healthcare access processes, geographic barriers, discharge logistics) that operate through different pathways to influence health outcomes [11]. In our study, age and race/ethnicity represent demographic characteristics that intersect with social drivers but do not themselves constitute structural drivers of health. Insurance type serves as a validated proxy measure for socioeconomic position, while discharge disposition reflects both individual social circumstances and structural healthcare system factors affecting post-acute care coordination. For this study, we specifically examined key social drivers including age, race/ethnicity, socioeconomic status (insurance type), geographic accessibility (distance to clinic), and post-discharge living arrangement (discharge disposition). These factors were selected based on prior literature demonstrating their impact on healthcare access and treatment adherence in chronic disease populations, and their modifiability through targeted healthcare system interventions.

Patients who fail to comply with outpatient follow-up care for their chronic wounds are often re-hospitalized for complications arising from untreated wounds. It has been previously concluded that 19.5% of Medicare patients will be readmitted to the hospital within 30 days of discharge [6]. Patients suffering from chronic wounds who do not seek appropriate care in a timely manner are at a risk of being readmitted. This study evaluated the 90 day readmission rates for chronic wound patients who did and did not follow-up as outpatients in a tertiary wound care clinic post-hospitalization.

## 2. Materials and Methods

This retrospective cohort study was conducted at a single tertiary care center within the Northwell Health system. As a single-center study, generalizability of findings may be limited to similar tertiary care settings with comparable patient demographics and standardized wound care protocols. Due to the retrospective nature of the study, the requirement for informed consent was waived.

**Figure 1:**
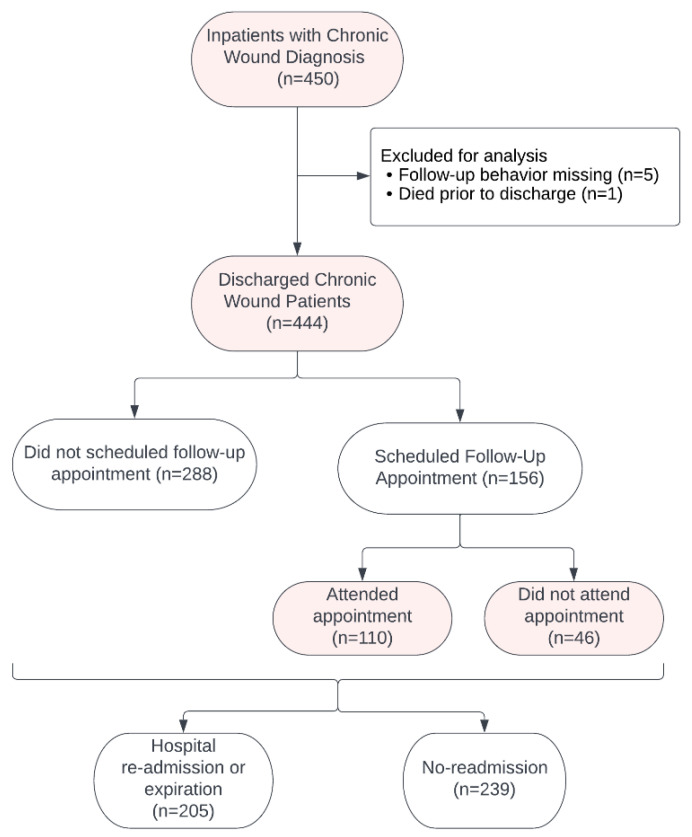
Study enrollment flowchart for chronic wound patients acquired from inpatient admission and subsequent follow-up adherence behavior.

A total of 450 inpatient medical charts were screened for inclusion based on documentation of an inpatient wound care consultation between August 2017 and June 2020, regardless of primary admission diagnosis. Patients were excluded if (1) follow-up appointment decision information was not recorded in the medical record (n = 5), or (2) the patient expired prior to inpatient discharge (n = 1), resulting in a final analytical cohort of 444 patients (Figure 1).

Patient demographic, clinical, and follow-up data were systematically extracted from the hospital’s electronic medical record system. Information was captured from hospital discharge summaries, inpatient wound care consultation notes, outpatient appointment records, and subsequent hospital readmission documentation.

Demographic variables collected included age, sex, self-reported race (categorized as White, Black/African American, Asian, or Other), and ethnicity (Hispanic or non-Hispanic). Age was analyzed both as a continuous variable and categorized into clinically relevant groups (<65 years, 65–79 years, ≥80 years).

Pre-discharge appointment scheduling was performed by nursing staff and case managers during the discharge planning process, with scheduled appointments documented in the electronic medical record. The scheduling decision represented a complex interplay of patient and family agreement, clinical team recommendations, discharge destination, and anticipated post-discharge care coordination capabilities rather than solely patient-level choice. For patients discharged to skilled nursing facilities, outpatient follow-up attendance required SNF staff coordination with medical transportation, as the Northwell Health system does not provide inter-facility transportation services. This represents a significant structural barrier independent of patient preference or clinical need, as SNF patients typically do not control their own outpatient scheduling or transportation logistics. The term “scheduled appointment” in this study refers to documented appointment dates in the medical record system, indicating that discharge planning personnel initiated the scheduling process, though this does not ensure patient awareness or ability to attend.

### 2.1. Clinical Variables and Comorbidity Assessment

Clinical variables included primary wound etiology, classified as pressure ulcer (PU), venous leg ulcer, diabetic foot ulcer (DFU), trauma, surgical, or other/unknown. One patient was excluded from wound source analysis due to missing classification data (n = 443 for wound-specific analyses). Comorbidity burden was quantified using the Charlson Comorbidity Index (CCI), a validated prognostic tool that assigns weighted scores to 19 medical conditions based on their association with one-year mortality risk. CCI scores were calculated from documented diagnoses in the EMR, with possible scores ranging from 0 to 16. Individual comorbidities, particularly congestive heart failure given its known association with wound healing impairment, were also analyzed separately. Additional clinical factors included length of hospital stay (measured in days from admission to discharge) and type of health insurance coverage, categorized as: (1) Medicaid (patients qualifying for Medicaid regardless of other coverage), (2) Medicare only (patients with Medicare without Medicaid qualification), or (3) private insurance (patients with private insurance without Medicaid qualification). Additional social drivers including educational attainment, primary language, and household income were not systematically documented in the electronic medical record system. We used insurance type as a validated proxy for socioeconomic position based on its correlation with income eligibility thresholds in prior health services research. Race and ethnicity data served as partial indicators of potential language or cultural barriers, though primary language was not directly assessed.

### 2.2. Statistical Analysis

Descriptive statistics were calculated for all variables. Continuous variables were assessed for normality using the Shapiro–Wilk test. Non-normally distributed continuous variables are presented as median with interquartile range (IQR) and compared using the Mann–Whitney U test or Kruskal–Wallis test as appropriate. Categorical variables are presented as frequencies and percentages and compared using chi-square tests or Fisher’s exact test when expected numbers were less than five. Logistic regression was used to identify factors associated with appointment scheduling and attendance, with results reported as odds ratios (OR) with 95% confidence intervals (CI). Variables with *p* < 0.10 in univariate analysis were considered for inclusion in multivariate models.

Time to hospital readmission or death was analyzed using Kaplan–Meier survival curves, with group comparisons performed using the log-rank test. Cox proportional hazards regression was used to assess the association between follow-up care receipt and 90-day readmission/mortality, adjusting for potential confounders including age, sex, race, ethnicity, CCI score, and congestive heart failure diagnosis. Results are reported as unadjusted hazard ratios (HR) and adjusted hazard ratios (aHR) with 95% CI. All statistical tests were two-tailed, and *p*-values < 0.05 were considered statistically significant. All analyses were performed using GraphPad Prism Version 9.4.1 (458).

## 3. Results

### 3.1. Demographic Factors Associated with Follow-Up Appointment Scheduling

Of the 444 patients included in the study, 156 (35.1%) scheduled an outpatient follow-up appointment prior to hospital discharge. Both patients who scheduled and did not schedule appointments were predominantly non-Hispanic (91.0% vs. 93.4%, respectively). The median Charlson Comorbidity Index (CCI) score was comparable between the two groups at 4 out of 16 (Table 1). However, significant demographic differences emerged in age and race. Patients who did not schedule a follow-up appointment were significantly older (median age 79 years [IQR, 64–89]) compared to patients who did schedule an appointment (median age 70 years [IQR, 58–79], *p* < 0.0001). Additionally, patients who did not schedule appointments were more likely to be of Asian descent (5.2% vs. 1.3%, *p* = 0.047). However, the absolute number of Asian patients was small (n = 17 total, representing 3.8% of the cohort), limiting the statistical power and generalizability of this finding. When stratified by clinically relevant age groups, patients aged 80 years or older were significantly less likely to schedule an appointment (OR, 0.42; 95% CI, 0.25-0.71; *p* = 0.0015). Clinically, this age-related decline in scheduling behavior suggests that elderly patients may face barriers in comprehension, decision-making capacity, or willingness to commit to additional medical appointments during the discharge process.

### 3.2. Appointment Attendance Among Scheduled Patients

Of the 156 patients who scheduled outpatient follow-up appointments, 110 (70.5%) attended their scheduled visit. Notably, age and racial identity, which had been significantly different between patients who scheduled versus did not schedule appointments, were comparable between patients who attended versus did not attend their scheduled appointments. The median age for patients who attended was 70 years [IQR, 59–81] compared to 69 years [IQR, 55–79] for those who did not attend (Table 2). Both groups were predominantly White (81.8% and 91.3%, respectively) and non-Hispanic (89.1% and 60.8%, respectively). There was no difference in comorbidity burden between the two groups, with both sharing a median CCI score of 4 (Table 2).

### 3.3. Ninety-Day Hospital Readmission and Mortality

Of the 444 discharged patients, 205 (46.2%) were readmitted to the hospital or expired within 90 days (Table 3). The median age for readmitted or expired patients was comparable to non-readmitted patients (74 years [IQR, 61–84] vs. 76 years [IQR, 63–89]). Both readmitted or expired and non-readmitted patients were predominantly White (63.9% vs. 67.8%, respectively) and non-Hispanic (93.2% vs. 92.1%, respectively), with no significant racial or ethnic differences between groups. However, patients who were readmitted or expired were significantly more likely to be male (57.6% vs. 48.1%; OR, 1.5; 95% CI, 1.0–2.12; *p* < 0.05) and had a higher median CCI score (4 [IQR, 3–6] vs. 4 [IQR, 2–5], *p* < 0.001). Further analysis revealed that patients with congestive heart failure were significantly more likely to be readmitted or expired within the 90-day timeframe (OR, 1.7; 95% CI, 1.15-2.57; *p* = 0.0097).

### 3.4. Clinical Characteristics and Their Impact on Follow-Up Behavior

#### 3.4.1. Length of Hospital Stay

Clinical characteristics of chronic wound patients, including length of hospital stay and wound source, were examined across all three endpoints. One patient was excluded from wound source analysis due to missing information (n = 443). Patients who scheduled a follow-up appointment before discharge had a significantly shorter median hospital stay of 6 days [IQR, 3–11] compared to patients who did not schedule a follow-up (9 days [IQR, 5–15.5], *p* < 0.0001). Among the 156 patients who scheduled follow-up appointments, the median hospital stay length for patients who attended their appointments (6 days [IQR, 3.8–11]) was comparable to those who did not attend (6 days [IQR, 3.0–11.3]). Patients who were readmitted to the hospital or expired had a median length of stay of 8 days [IQR, 4–15] compared to 7 days [IQR, 5–14] for non-readmitted patients. The association between prolonged hospitalization and lower scheduling rates may reflect patient fatigue, diminished receptivity to discharge instructions, or more complex medical conditions that complicate post-discharge planning.

#### 3.4.2. Wound Etiology

The primary source of chronic wound development varied across the patient cohort. Among patients examined for scheduling follow-up appointments, pressure ulcers were the most common etiology (40.4%), followed by venous leg ulcers (30.0%) (Table 1). This pattern was consistent among patients examined for appointment attendance (33.6% pressure ulcers and 34.5% venous leg ulcers, respectively) and for hospital readmission (40.4% and 30.0%, respectively). Other wound sources included trauma, surgical wounds, diabetic foot ulcers (DFU), and other or unknown etiologies. Using pressure ulcer cases as the reference category, odds ratio analysis revealed that patients with diabetic foot ulcers were significantly more likely to schedule a follow-up appointment (OR, 4.5; 95% CI, 1.62–12.50; *p* = 0.004). No other wound sources were found to significantly impact appointment attendance or 90 day readmission rates (Table 2 and Table 3).

### 3.5. Social Drivers of Health

#### 3.5.1. Discharge Destination

Chronic wound patients were discharged from the hospital to either a skilled nursing facility or home. Discharge destination significantly influenced follow-up scheduling behavior, with appointments being significantly more likely to be scheduled among patients discharged home (73.7%) compared to those discharged to a skilled nursing facility (26.3%; OR, 0.4; 95% CI, 0.26–0.60; *p* < 0.0001). Among patients who scheduled follow-up appointments, those discharged home (23.5%) were significantly more likely to attend their outpatient appointment than patients discharged to a skilled nursing facility (19.0%; OR, 3.3; 95% CI, 1.56–6.92; *p* < 0.01). However, discharge destination did not have a significant association with 90 day hospital readmission rates. The finding that skilled nursing facility residents were less likely to both schedule and attend appointments suggests systemic barriers in care coordination and transportation logistics for this vulnerable population.

#### 3.5.2. Health Insurance Type

The type of health insurance coverage was not a determining factor for wound care follow-up adherence or hospital readmission outcomes. Healthcare insurance was categorized into three groups: Medicaid (including all Medicaid-qualifying patients regardless of other coverage), Medicare only (patients with Medicare without Medicaid qualification), and private insurance (patients with private insurance without Medicaid qualification). Of the total 383 patients with documented insurance information for scheduling analysis, the majority were categorized under Medicare (38.6%) or Medicaid (36.2%). Insurance type did not affect patients’ scheduling of follow-up appointments (Table 1). Among the 138 patients with insurance information who scheduled appointments, most had private insurance (36.7%) or Medicaid (35.7%) (Table 2). For patients analyzed for hospital readmission, most had Medicare (36.2%) or Medicaid (38.6%) (Table 3). Using Medicaid as the reference category for all assessed conditions (Table 1, Table 2 and Table 3), odds ratio analysis did not determine a significant association between insurance type and appointment scheduling, attendance, or hospital readmission.

#### 3.5.3. Geographic Distance to Clinic

Distance from the patient’s care setting to the outpatient clinic was examined as a potential barrier to receiving follow-up care. The median distance for patients who scheduled a follow-up appointment (8.8 miles [IQR, 5.5–13.5]) was comparable to patients who did not schedule an appointment (8.9 miles [IQR, 5.1–12.6]) (Table 1). Similarly, the median distance for patients who attended their scheduled appointments (9.9 miles [IQR, 6–13.5]) was comparable to patients who did not attend (11.0 miles [IQR, 7.4–14.8]) (Table 2). The median distance for patients who were readmitted to the hospital or expired within 90 days (8.8 miles [IQR, 5.1–12.9]) was also comparable to patients who were not readmitted (9.0 miles [IQR, 5.4–12.9]) (Table 3). Statistical analysis using unpaired parametric t-tests for all measures did not identify geographic distance to the clinic as a significant factor influencing any of the study outcomes.

### 3.6. Impact of Follow-Up Attendance on Ninety-Day Hospital Readmission

Attendance at outpatient wound care follow-up appointments was significantly associated with 90 day hospital readmission rates. The process of acquiring follow-up care consisted of two sequential steps: scheduling a follow-up appointment before hospital discharge (Table 1) and subsequent patient attendance at the scheduled appointment (Table 2). Analysis revealed that merely scheduling a follow-up appointment did not significantly impact 90 day readmission or mortality (OR, 1.1; 95% CI, 0.76–1.67; *p* = 0.55). However, patients who actually received outpatient follow-up care were significantly less likely to be hospitalized or expire within the 90 day period (OR, 0.3; 95% CI, 0.15–0.66; *p* = 0.002) (Table 4).

**Figure 2:**
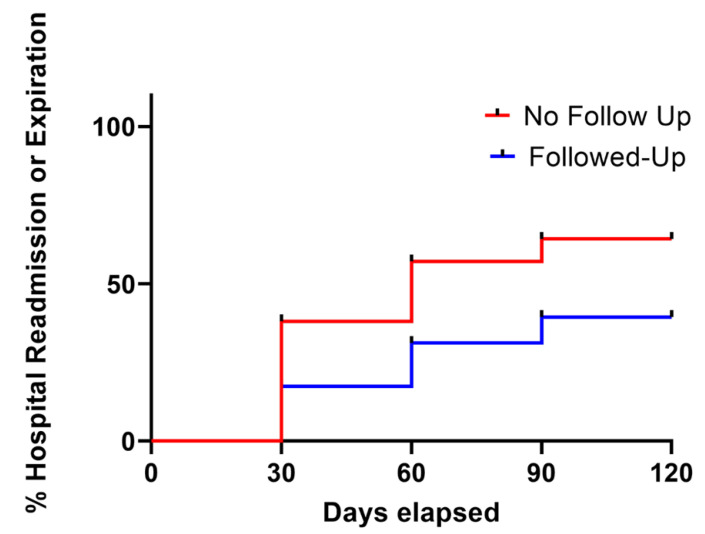
Kaplan–Meier survival curve analysis of patients with follow-up and no follow-up 90 days after hospital discharge, assessing the percentage not hospitalized or expired over 30-day increments (hazard ratio, 2.58, 95% CI, 1.440–4.75). Patients not hospitalized post-90 days were considered not hospitalized or expired within this study.

Kaplan–Meier survival curve analysis comparing patients who received outpatient wound care follow-up versus those who did not receive follow-up care was consistent with the odds ratio analysis. The hazard ratio demonstrated a significantly higher risk of hospitalization or death in patients without follow-up care (HR, 2.58; 95% CI, 1.40–4.75; log-rank test, χ^2^ = 9.28; *p* = 0.002) (Figure 2). After adjusting for age, sex, race, ethnicity, CCI score, and congestive heart failure diagnosis, Cox multivariate regression analysis similarly determined that lack of follow-up care was associated with a significantly higher risk of readmission or death (aHR, 2.39; 95% CI, 1.45–3.89; *p* < 0.001). These findings underscore the clinical importance of ensuring patients not only schedule but attend wound care follow-up appointments, as scheduling alone conferred no benefit.

## 4. Discussion

In this retrospective cohort study of discharged, chronic wound patients, we identified the timepoints by which social drivers of health and clinical factors were associated with receiving clinically significant outpatient follow-up care, recognizing that scheduling behavior reflects both patient-level and system-level factors. Pre-discharge scheduling represents the primary barrier to follow-up adherence, with patients ≥80 years old and those discharged to skilled nursing facilities substantially less likely to schedule appointments. Among patients who scheduled appointments, majority of patients attended, demonstrating that the scheduling decision itself is the critical determinant. Moreover, actual receipt of outpatient wound care reduced 90 day readmission or mortality risk by more than two-fold, independent of age, comorbidities, and other confounders. These findings suggest that targeted communication interventions at hospital discharge, particularly for elderly patients and skilled nursing facility residents, represent a high-yield strategy for improving wound care outcomes.

An increasing number of recent literatures [7] have demonstrated evidence-based guidelines for optimizing chronic wound outcomes; however, administering of improved wound care guidelines has remained at a sub-optimal level [8,9]. To determine patient-side factors affecting wound care management, three endpoints were examined following hospital discharge, including follow-up scheduling, follow-up adherence, and hospital readmission within a 90 day window.

Our results are consistent with prior studies correlating standardized, interdisciplinary wound management with improved patient outcomes. The introduction of evidence-based practice in treating lower extremity ulcers has been significantly associated with marked improvement in healing outcomes [7] with some studies reporting as high as a 62% improvement [9]. Prior literature also underscores the importance of continuing chronic wound care under the supervision of multidisciplinary staff, provided the multi-system-dependent nature of wound healing [2,12,13,14]. Similarly, this study implemented a workflow streamlining the referral of chronic wound inpatients into the care of a multidisciplinary staff with the measured endpoint of 90 day hospital readmission. Based on OR and Kaplan–Meier survival analysis, patients who received follow-up wound management were significantly less likely to be readmitted. Controlling for additional confounding factors (e.g., age, sex race, ethnicity, comorbidities), Cox-multivariate regression revealed that the incidence of readmission or expiration across 90 days for patients who did not receive follow-up care was 2.4-fold higher than those who received care. As seen in previous wound care follow-up studies, the implementation of this standardized, outpatient care protocol is an independent predictor for improving chronic wound patient outcomes [9,13,15,16].

The latency between discharge to follow-up could be a factor of interest in hospital readmission rates for chronic wound patients. While not statistically significant within this study, timeliness to follow-up has been established as a key strategy in reducing hospital readmission in several studies, noting improved patient outcomes in transitioning to permanent dispositions [16]. In the cases of wound care, continued management of wound progression is important to assess whether initial assessment and wound bed preparation has been efficacious, especially for elderly or disabled patients lesser abled to self-maintain a proper healing environment. Time-sensitive interventions, such as revascularization in peripheral arterial disease patients and improved perfusion of lower extremity wounds, can significantly improve healing outcomes when performed earlier on [17]. Clarity on this factor would require further investigation into primary readmission diagnoses with larger sample size.

### 4.1. Communication Interventions During Discharge

This study identifies an important timepoint to close the gap between trial-reported and real-world patient reception of standardized, multidisciplinary chronic wound care. Our data quantitatively shows that majority of patients that scheduled a follow-up adhered to their appointment. Social drivers of health and hospitalization appeared to play a larger role at the scheduling stage of follow-up adherence, potentially attributed to the difficulty in reaching out to patients post-discharge. Our patient sample included all hospitalized patients with chronic wounds at a tertiary hospital who sought out an in-house wound care staff. We reasoned that the inclusion of a broader sample was more likely to recognize previously unidentified at-risk wound patients for follow-up adherence. Provided the critical gap between real-world outcomes and those seen receiving standardized, interdisciplinary care in trials, we sought to identify these at-risk groups to better provide targeted intervention for encouraging timely follow-up care.

Scheduling an outpatient wound care follow-up at discharge was deemed the initial endpoint of follow-up adherence in this study. Breakdown of patients into clinically relevant age groups [17] found patients older than 80 years old to be the threshold for significantly lower rates of scheduling follow-up. Our data demonstrates that patients ≥80 years were significantly less likely to schedule appointments. However, the mechanisms underlying this association were not directly evaluated. Potential explanations include age-related cognitive decline affecting comprehension, diminished health literacy in older populations, or caregiver availability limitations. Although not evaluated, many of these patients may face personal difficulties negatively affecting their willingness to schedule an appointment. For example, comprehension of continued outpatient wound management should be assessed in patients. Mental acuity assessment in elderly patients often overlooked, resulting in failure to understand the importance of their follow-up appointment [17]. Alternative strategies to support understanding of follow-up importance should be implemented. In cases where cognitive difficulty hinders the clinical decision-making process, family education has been highlighted for adherence to discharge instructions and therapeutic regimen due to greater social support or assistance with transportation accessibility. Misinterpretation of outpatient services can also negatively affect specific ethnicities. We observed that Asian patients were less likely to schedule follow-up appointments, though the small sample size limits interpretation. Without data on primary language, English proficiency, health literacy, or socioeconomic factors specific to this subgroup, we cannot determine whether this finding reflects language barriers, cultural factors, socioeconomic differences, or unmeasured confounders. Prior research has documented that Asian ethnic subgroupsshowed hesitancy towards Western healthcare based on language or cultural attitude barriers, [18] though we could not directly assess language proficiency or cultural attitudes in this retrospective study. Compounding limited English proficiency and cultural background in older Asian populations can increase the challenge of understanding the nuanced explanation of wound care. The scarcity of wound care education resources remains lacking for the general public, particularly in non-English languages, causing greater difficulty for patients to recognize the need for professional assistance for a non-healing wound. Prior systematic reviews and observational studies have previously demonstrated how communication interventions are highly correlated with reducing hospital readmission, reasons attributed to low adherence to following instructions or treatment regimens [19,20,21]. Educational interventions on chronic disease-specific education have been implicated to be a major factor in adherence to treatment regimen [22]. In the case of wound care management, visualization through digital videos or step-by-step manual instructions may provide a clearer understanding of the needs of wound management, and extensive conversation with family members may be necessary to communicate the importance of continued wound care follow-up. This highlights the need for providers to take lifestyle barriers and sociocultural considerations in ensuring comprehension of the reasons for continued wound care management.

Patients with a prolonged duration of initial hospitalization at 9 compared to 6 days were reported with significantly lower appointment scheduling rates. During the discharge process, providers are expected to provide multiple pieces of clinically relevant information, including diagnoses, treatment, and in the interest of chronic conditions such as chronic wounds, follow-up care. We observed that patients with longer hospitalizations were significantly less likely to schedule follow-up appointments. While our study did not assess patient psychological state or receptivity, prior research suggests that prolonged hospitalization may cause mental exhaustion that decreases patient receptivity towards a clinical setting, whereas a prolonged length of stay is likely to exacerbate aversion [22]. Reasons to consider for this study may be the elderly population being more susceptible to mental tiredness from a prolonged stay in a hospital environment, due to the inconvenience and discomfort from the change in living habits. Survey reports of hospitalized geriatric patients that were discharged commonly reported an extended time away from home motivated the return to familiar comforts and emotional exhaustion, or competing priorities at discharge remain unclear and warrants further investigation [23,24,25]. Willingness and acuity to regard provider explanation on continued wound care management may be substantially varied approaching discharge. While the initial hospitalization diagnoses may have been resolved before discharge, patient concerns are likely to prioritize returning to their original quality of living than chronic wound management. Thus, earlier discussion and repetitive reinforcement of the importance of continued chronic wound management post-discharge throughout the length of stay may improve patient comprehension. Given the emotional and mental fatigue after prolonged hospitalization, this will potentially improve patient agreeability and greater understanding of the dangers posed by unmanaged wounds.

A significant social determinant of health associated with both follow-up scheduling and impacting follow-up adherence was patient disposition. Odd’s ratio analysis reported skilled nursing facility (SNF) patients being 61% and 70% less likely to schedule and of those, attend their wound care follow-up, respectively, than at-home patients. The lower scheduling and attendance rates among SNF-bound patients likely reflect structural barriers in care coordination rather than individual patient preferences. SNF patients face institutional constraints including resource allocation issues faced by skilled nursing facilities and lack of control over outpatient scheduling and transportation logistics [24]. For example, the 2014 Medicare Skilled Nursing Facility Value-Based Purchasing program introduced financial incentives based on 30 day hospital readmission rates, to which already struggling SNFs reeled further from resource assistance [324. Structural and internal SNF infrastructure are forced to distribute resources across their patient population, rather than pertinent transportation for individual chronic wound patients. Thus, individualized outpatient transport for differing dates and locations may prove unsustainable. Coordination with SNFs should be established to prevent sole handling of logistical issues by the patient to better streamline follow-up. In communication, options such as batching transport for patients with a close range of follow-up appointments or developing transitional care models to identify wound interventions with the greatest need for follow-up would enable optimal resource allocation.

### 4.2. Limitations

There are inherent limitations to using 90 day hospital readmission or expiration as a measure of wound care follow-up efficacy. Identifying shortcomings and timing of patient education at hospital discharge has been previously associated with increased risk of treatment failure and hospital readmission [25, which forms the basis of this study focusing on the timing of targeted interventions for at-risk groups within a chronic wound population. Given that reason for readmission or expiration was not recorded, this endpoint does not serve as a direct measure of wound prognoses, but rather patient outcomes. Apart from chronic wound complications, compounding comorbidities accompanying this aging population could lead to hospitalization from life-threatening complications or non-urgent lifestyle discomfort [6]. Important social drivers of health including educational attainment and household income were not systematically documented in the electronic medical record system and therefore could not be retrospectively evaluated. The absence of primary language data limits our ability to directly assess language barriers as a contributor to follow-up non-adherence, though race and ethnicity served as partial indicators of potential cultural or linguistic challenges. We used insurance type as a validated proxy measure for socioeconomic status, though this does not capture the full complexity of economic barriers patients may face.

Selection bias may exist as our cohort included only hospitalized patients receiving wound care consultations, potentially excluding less severe cases managed in outpatient settings. Patients who scheduled appointments may inherently differ in health-seeking behaviors or social support, representing unmeasured confounding. Information bias is inherent to the retrospective design, as we could not assess the content or quality of discharge conversations, and reasons for not scheduling appointments (patient refusal vs. not offered vs. institutional barriers) were not documented. Social drivers of health not assessed in this study, such as educational level and economic status, could also lower the decision threshold for hospital readmission. Additional factors that were identified as significant (e.g., DFU patients in scheduling, male patients being more prone to follow-up) and factors of interest that almost crossed the significance threshold (e.g., patient’s insurance) had limited sample sizes that may reflect insufficient statistical power rather than true null effects. Specifically, the small number of Asian patients (n = 17, 3.8% of cohort) and diabetic foot ulcer patients (n = 18) limits generalizability and definitive conclusions for these subgroups.

## 5. Conclusions

The findings of this retrospective review indicate that social drivers of health and hospital stay are associated with reducing scheduling rates of wound care follow-up, and skilled nursing facility disposition reduces adherence to scheduled appointments. The pre-discharge process in communicating follow-up scheduling is a crucial time point for wound treatment adherence, where certain patient groups are more susceptible to personal and social circumstances discouraging outpatient follow-up. Communication interventions tailored towards at-risk groups during hospital discharge may improve patient comprehension of outpatient wound management. Thus, healthcare systems should focus on tailored communication approaches at discharge to improve chronic wound patient outcomes.

Future research should prospectively evaluate the effectiveness of targeted discharge interventions for high-risk populations, including structured cognitive assessments for elderly patients, culturally tailored multilingual education materials, and systematic family involvement in discharge planning. Implementation studies are needed to test care transition models that coordinate transportation and appointment scheduling for skilled nursing facility residents. From a health policy perspective, our findings support reimbursement models that incentivize pre-discharge care coordination and transitional care management for chronic wound patients. Healthcare systems should consider developing standardized discharge protocols that identify at-risk patients and allocate additional resources for discharge education and follow-up facilitation. Given the increased risk of readmission without follow-up care, investments in discharge communication interventions represent a potentially high-value strategy for reducing healthcare costs while improving patient outcomes.

## Figures and Tables

**Table 1:** Demographic and clinical factors associated with patient setting up a follow-up appointment immediately after inpatient discharge for chronic wound complications.

Factor	Total Sample (n = 444)	Appointment Made (n = 156)	Appointment Not Made (n = 288)	OR [95% CI]	*p*-Value
Age, years					<0.0001
Median (Q1, Q3)	75 (62, 86)	70 (58, 79)	79 (64, 89)	-	
Age Group					
0–19	1 (0.2)	(0)	1 (0.3)	inf.	-
19–45	33 (7.4)	15 (9.6)	18 (6.2)	0.7 [0.35–1.61]	0.44
45–65	109 (24.5)	49 (31.4)	60 (20.8)	0.8 [0.46–1.23]	0.26
65–80	171 (38.5)	65 (41.6)	106 (36.8)	ref.	
80+	130 (29.2)	27 (17.3)	103 (35.7)	0.4 [0.25–0.71]	0.002
Sex,					0.24
Male, n (%)	233 (52.48)	76 (48.7)	157 (54.5)	0.8 [0.53–1.18]	
Female, n (%)	211 (47.52)	80 (51.2)	131 (45.4)	-	
Race					
White, n (%)	293 (65.99)	104 (66.7)	189 (65.6)	ref.	
Black, n (%)	75 (16.89)	30 (19.2)	45 (15.63)	1.2 [0.72–2.03]	0.47
Asian, n (%)	17 (3.83)	2 (1.3)	15 (5.2)	0.2 [0.05–0.91]	0.04
Other, n (%)	59 (13.29)	20 (12.8)	39 (13.5)	0.9 [0.52–1.70]	0.81
Ethnicity					0.36
Hispanic, n (%)	33 (74.3)	14 (8.9)	19 (6.6)	1.4 [0.67–2.91]	
Not Hispanic, n (%)	411 (92.57)	142 (91.0)	269 (93.4)	-	
Charlson Comorbidity Index					0.28
Score, median (Q1, Q3)	4 (3, 5)	4 (2, 5)	4 (3, 5)	-	
Wound Type ^a^					
PU, n (%)	179 (40.41)	55 (35.3)	124 (43.2)	ref.	
Venous, n (%)	133 (30.0)	55 (35.2)	78 (27.2)	1.6 [1.01–2.52]	0.06
Trauma, n (%)	38 (8.6)	9 (5.7)	29 (10.1)	0.7 [0.30–1.61]	0.44
Surgical, n (%)	33 (7.4)	11 (7.0)	22 (7.7)	1.1 [0.52–2.42]	0.83
DFU, n (%)	18 (4.1)	12 (7.6)	6 (2.1)	4.5 [1.62–12.50]	0.004
Other, n (%)	42 (9.5)	14 (8.9)	28 (9.7)	1.1 [0.54–2.26]	0.85
Hospital length of stay,					<0.0001
Days, median (Q1, Q3)	7.5 (4.0, 4.0)	6.0 (3.0, 11.0)	9.0 (5.0, 15.5)	-	
Care Setting					
Skilled nursing facility, n (%)	178 (40.1)	41 (26.3)	137 (47.6)	0.4 [0.26–0.60]	<0.0001
Home, n (%)	266 (59.9)	115 (73.7)	151 (52.4)	-	
Health insurance ^b^, n (%)					
Medicaid	148 (38.6)	54 (39.1)	94 (38.3)	ref.	-
Medicare	139 (36.2)	40 (28.9)	99 (40.4)	0.7 [0.43–1.17]	0.17
Private, n (%)	96 (25)	44 (31.8)	52 (21.2)	1.6 [0.94–2.64]	0.18
Distance to clinic,					0.34
Miles, median (Q1, Q3)	8.8 (5.3,12.9)	8.8 (5.5,13.5)	8.9 (5.1,12.6)	-	

^a^ Type of wound information missing for one patient (n = 443). ^b^ Medicaid group included all patients with Medicaid, Medicare patients included patients possessing Medicare with or without supplemental private insurance, and private insurance patients included all other insurances; totaling n = 383.

**Table 2:** Demographic and clinical factors associated with patients attending follow-up appointments, among patients that previously set up an appointment.

Factor	Attended Appointment (n = 110)	Did Not Attend Appointment (n = 46)	OR [95% CI]	*p*-Value
Age				>0.999
Years, median (Q1, Q3)	70 (59, 81)	69 (55, 79)	-	
Sex, n (%)				>0.999
Male	56 (50.9)	24 (52.1)	1.0 [0.48–1.88]	
Female	54 (49.1)	22 (47.8)	-	
Race, n (%)				
White	76 (69)	28 (60.8)	ref.	
Black	20 (18.1)	10 (21.7)	0.7 [0.32–1.80]	0.49
Asian	2 (1.8)	0 (0)	inf.	0.39
Other	12 (10.9)	8 (17.3)	0.6 [0.20–1.53]	0.24
Ethnicity, n (%)				0.36
Hispanic	9 (8.1)	5 (10.8)	0.7 [0.23–2.05]	
Not Hispanic	101 (91.8)	41 (89.1)	-	
Charlson Comorbidity Index				>0.999
Score, median (Q1, Q3)	4 (2, 5)	4 (3, 5.3)	-	
Wound Type ^a^, n (%)				
PU	37 (33.6)	18 (39.1)	ref.	
Venous	38 (34.5)	17 (36.9)	1.1 [0.51–2.34]	>0.999
Trauma	9 (8.1)	3 (6.5)	1.5 [0.38–5.46]	0.74
Surgical	8 (7.2)	0 (0)	inf.	-
DFU	7 (6.3)	5 (10.8)	0.7 [0.19–2.28]	0.74
Other	11 (10)	3 (6.5)	1.8 [0.50–6.52]	0.53
Length of Stay				0.63
Days, median (Q1, Q3)	6.0 (3.8, 11.0)	6.0 (3.0, 11.3)	-	
Care Setting, n (%)				<0.01
Skilled nursing facility	21 (19)	20 (43.4)	0.3 [0.14–0.65]	
Home	89 (23.5)	26 (76.9)	-	
Health insurance ^b^, n (%)				
Medicaid	35 (35.7)	19 (47.5)	ref.	
Medicare	27 (27.5)	13 (32.5)	1.1 [0.47–2.54]	0.83
Private	36 (36.7)	8 (20)	2.4 [0.93–6.30]	0.07
Distance to clinic,				0.70
Miles, median (Q1, Q3)	9.9 (6.0,13.5)	11.0 (7.4, 14.8)	-	

^a^ Type of wound information missing for one patient (n = 443). ^b^ Medicaid group included all patients with Medicaid, Medicare patients included patients possessing Medicare with or without supplemental private insurance, and private insurance patients included all other insurances (n = 138).

**Table 3:** Demographic and clinical factors associated with patient re-admission into the hospital or expiration within 90 days of discharge for chronic wound complications.

Factor	Total Sample (n = 444)	Re-Admission or Expired (n = 205)	No Re-Admission (n = 239)	OR [95% CI]	*p*-Value
Age					0.14
Years, median (Q1, Q3)	75 (62, 86)	74 (61, 84)	76 (63, 89)	-	
Sex, n (%)					0.047
Male	233 (52.5)	118 (57.6)	115 (48.1)	1.5 [1.00–2.12]	
Female	211 (47.5)	87 (42.4)	124 (51.9)	-	
Race, n (%)					
White	293 (66.0)	131 (63.9)	162 (67.8)	ref.	
Black	75 (16.9)	35 (17.1)	40 (16.7)	1.1 [0.64–1.80]	0.76
Asian	17 (3.8)	10 (4.9)	7 (2.9)	1.8 [0.63–4.57]	0.26
Other	59 (13.3)	29 (14.1)	30 (12.6)	1.2 [0.70–2.06]	0.53
Ethnicity, n (%)					0.65
Hispanic	33 (7.4)	14 (6.8)	19 (7.9)	0.8 [0.41–1.76]	
Not Hispanic	411 (92.6)	191 (93.2)	220 (92.1)	-	
Charlson Comorbidity Index					<0.001
Score, median (Q1, Q3)	4 (3, 5)	4 (3, 6)	4 (2, 5)	-	
CHF	135	75	60	1.7 [1.15–2.57]	0.01
No CHF	309	130	179	-	
Wound Type^a^, n (%)					0.09
PU	179 (40.4)	80 (39.2)	99 (41.4)	ref.	-
Venous	133 (30.0)	68 (33.3)	65 (27.2)	1.3 [0.83–2.03]	0.30
Trauma	38 (8.6)	11 (5.4)	27 (11.3)	0.5 [0.23–1.07]	0.10
Surgical	33 (7.5)	16 (7.8)	17 (7.1)	1.2 [0.55–2.43]	0.71
DFU	18 (4.1)	12 (5.9)	6 (2.5)	2.5 [0.90–6.81]	0.08
Other	42 (9.5)	17 (8.3)	25 (10.5)	0.8 [0.43–1.62]	0.73
Length of stay					0.66
Days, median (Q1, Q3)	8 (4, 14)	8 (4, 15)	7 (5, 14)	-	
Care Setting, n (%)					0.87
Skilled nursing facility	266 (59.9)	122 (59.5)	144 (60.3)	1.0 [0.67–1.41]	
Home	178 (40.1)	83 (40.5)	95 (39.7)	-	
Health insurance ^b^, n (%)					
Medicaid	148 (38.6)	71 (41)	77 (36.6)	ref.	
Medicare	139 (36.2)	55 (31.7)	84 (40)	0.7 [0.45–2.24]	0.16
Private	96 (25)	47 (27.1)	49 (23.3)	1.0 [0.62–1.75]	0.89
Discharge to follow-up Latency,					
Distance to clinic,					0.76
Miles, median (Q1, Q3)	8.8 (5.3, 12.9)	8.8 (5.1, 12.9)	9.0 (5.4, 12.9)	-	

^a^ Type of wound information missing for one patient (n = 443). ^b^ Medicaid group included all patients with Medicaid, Medicare patients included patients possessing Medicare with or without supplemental private insurance, and private insurance patients included all other insurances; totaling n = 383.

**Table 4:** Examination of follow-up adherence behavior on hospital readmission or expiration within 90 days post-discharge.

Factor	Total Sample (n = 444)	Re-Admission or Expired (n = 205)	No Re-Admission (n = 239)	OR [95% CI]	*p*-Value
Follow-up appointment made					
Yes	156 (35.1)	75 (36.6)	81 (33.9)	1.1 [0.76–1.67]	0.55
No	288 (64.9)	130 (63.4)	158 (66.1)	-	
Received follow-up care					
Yes	110 (24.8)	44 (58.6)	66 (81.4)	0.3 [0.15–66]	0.002
No	334 (75.2)	31 (41.3)	15 (18.5)	-	

## Data Availability

Due to patient privacy protections under the Health Insurance Portability and Accountability Act (HIPAA), the data presented in this study are available from the corresponding author upon reasonable request and with appropriate institutional approval, data use agreements, and ethics committee clearance.

## Abbreviations

The following abbreviations are used in this manuscript:

CCI	Charlson Comorbidity Index
CHF	Congestive Heart Failure
CI	Confidence Interval
DFU	Diabetic Foot Ulcer
EMR	Electronic Medical Record
HIPAA	Health Insurance Portability and Accountability Act
HR	Hazard Ratio
aHR	Adjusted Hazard Ratio
IQR	Interquartile Range
OR	Odds Ratio
PU	Pressure Ulcer
SNF	Skilled Nursing Facility
